# Comparing a Fully Automated Cephalometric Tracing Method to a Manual Tracing Method for Orthodontic Diagnosis

**DOI:** 10.3390/jcm11226854

**Published:** 2022-11-20

**Authors:** Ioannis A. Tsolakis, Apostolos I. Tsolakis, Tarek Elshebiny, Stefanos Matthaios, J. Martin Palomo

**Affiliations:** 1 Department of Orthodontics, School of Dentistry, Aristotle University of Thessaloniki, 541 24 Thessaloniki, Greece; 2 Department of Orthodontics, School of Dentistry, National and Kapodistrian, University of Athens, 157 72 Athens, Greece; 3 Department of Orthodontics, School of Dental Medicine, Case Western Reserve University, Cleveland, OH 44106, USA

**Keywords:** cephalometrics, tracing, digital, automatic, manual, artificial intelligence

## Abstract

Background: This study aims to compare an automated cephalometric analysis based on the latest deep learning method of automatically identifying cephalometric landmarks with a manual tracing method using broadly accepted cephalometric software. Methods: A total of 100 cephalometric X-rays taken using a CS8100SC cephalostat were collected from a private practice. The X-rays were taken in maximum image size (18 × 24 cm lateral image). All cephalometric X-rays were first manually traced using the Dolphin 3D Imaging program version 11.0 and then automatically, using the Artificial Intelligence CS imaging V8 software. The American Board of Orthodontics analysis and the European Board of Orthodontics analysis were used for the cephalometric measurements. This resulted in the identification of 16 cephalometric landmarks, used for 16 angular and 2 linear measurements. Results: All measurements showed great reproducibility with high intra-class reliability (>0.97). The two methods showed great agreement, with an ICC range of 0.70–0.92. Mean values of SNA, SNB, ANB, SN-MP, U1-SN, L1-NB, SNPg, ANPg, SN/ANS-PNS, SN/GoGn, U1/ANS-PNS, L1-APg, U1-NA, and L1-GoGn landmarks had no significant differences between the two methods (*p* > 0.0027), while the mean values of FMA, L1-MP, ANS-PNS/GoGn, and U1-L1 were statistically significantly different (*p* < 0.0027). Conclusions: The automatic cephalometric tracing method using CS imaging V8 software is reliable and accurate for all cephalometric measurements.

## 1. Introduction

Since Broadbent developed the imaging technique in 1931, cephalometry has been used to investigate growth, identify malocclusions, and create treatment plans as well as to assess the outcomes of those treatments. After tracing anatomical features and identifying landmarks on acetate paper for the lateral cephalogram analysis, measurements were taken with rulers and protractors. The entire process was laborious, time-consuming, prone to mistakes, and largely dependent on operator skill. Developments in computer software have mostly served to automate the cephalometric measurements, but the doctor must still manually pinpoint the appropriate landmarks [1,2]. Nowadays, the advancement of different computer-based technologies such as artificial intelligence and 3D printing is giving a new perspective to the everyday orthodontic practice [3,4,5].

Artificial intelligence (AI) is the ability of a technology to mimic human intelligence or make decisions that are effective and ethical by predetermined criteria. AI may now encompass many facets of contemporary culture thanks to advances in analytics techniques, computer power, and data availability. We can already see its effects in our everyday lives on a global scale. It filters content for social media, web searches, and consumer goods such as cameras, cellphones, tablets, and even autos. Machine learning is a prominent branch of artificial intelligence. Machine learning uses the statistical patterns of previously learned data to predict new data and circumstances. Machine learning needs to incorporate training data in order to work, therefore, training data are necessary for machine learning to function. With this method, the computer model can learn from experience rather than through traditional explicit programming, improving over time. In order to learn the features of the data using abstractions from several processing levels, a model must be given a lot of data. Deep learning has the advantage that it does not require a lot of engineering work to preprocess the data and deep learning techniques have been employed most prominently in object identification and visual object recognition. Machine learning has become increasingly important in the detection and classification of specific diseases found in medical imaging as a result of recent technological advancements. There have been initiatives in orthodontics to use machine learning in various methods, one of which is the automated AI recognition of cephalometric landmarks. The two-dimensional cephalometric image is still the crucial and most often used tool in orthodontics for diagnosis, treatment planning, and result prediction even though research using three-dimensional imaging has garnered attention [6,7].

There have been numerous attempts to include AI in the cephalometric study. The International Symposium on Biomedical Imaging conferences, supported by the Institute of Electrical and Electronics Engineers, launched global AI challenges in 2014 for precise AI measurements. The challenge has changed since 2015, becoming more clinically focused and providing success categorization rates. The Institute of Electrical and Electronics Engineers (IEEE) and the International Symposium on Biomedical Imaging (ISBI) hosted challenges on the automatic recognition of cephalometric landmarks and presented 400 different lateral cephalograms. In addition, new algorithms have been created on the same open dataset. Some of these methods, including decision trees, random forests, and deep learning, have been used to increase the precision of landmark detection [8,9,10,11]. Additionally, the resultant point must be located on the average of the coordinates for the left and right landmarks when the bilateral anatomic features do not overlap. There has not been any research that has successfully described this ailment in the past. This situation may compromise the precision of landmark placement and compromise the reliability of the cephalometric study. The convolutional neural network (CNN), a deep learning network structure, is the algorithm that exhibits significant advantages in graphics processing. It has been applied to problems involving images such as target identification, character recognition, face recognition, posture assessment, and others. In medicine, and more specifically in medical imaging, CNN has been successfully used to detect and classify lesions, image segmentation, auxiliary diagnosis, etc. [12,13,14,15,16,17,18,19,20,21,22,23,24]. In 2019, Park et al., a year after Hwang et al., used two different kinds of CNN. The first one was “You-Only-Look-Once version 3” and the second one was the “Single Shot Multibox Detector”. They used both CNN types to find 80 landmarks with good results. Some of these landmarks were applied to perform measurement analysis, whereas others detected contours or outlines or were used to predict treatment outcomes [8,25].

Carestream dental is one of the companies that has shown high interest in automatic cephalometric tracing since the very beginning of this method. Nowadays, they offer a cephalometric imaging software capable of fully tracing any cephalometric X-ray taken by a Carestream cephalostat as long as the X-ray is taken in the maximum image size (18 × 24 cm lateral image). Cephalometric tracing is based on artificial intelligence, using a deep convolutional neural network (CNN) for landmark detection, followed by an active shape model (ASM) for adjusting the position of the whole structure. Some classical image processing (mathematical morphology) is also involved in tracing the soft-tissue profile. The software does not acquire information from the machine; the network is pre-trained. The training set was collected from a collaborator orthodontist, then manually annotated. There are many studies that looked over the accuracy of different software programs but, to the best of our knowledge, none of them looked over CS imaging V8 software (Carestream Dental LLC, Atlanta, GA, USA), which is a broadly used software. This study aims to compare the accuracy of automatic cephalometric analysis using CS imaging V8 software to manual cephalometric analysis.

## 2. Materials and Methods

Subjects were recruited from a private practice that owns a CS8100SC Evo Edition X-ray machine (Producer: Carestream Dental LLC, Atlanta, GA, USA, Year: 2020). Inclusion criteria consisted of subjects seeking orthodontic treatment whose records included cephalometric X-rays. Subjects with existing intraoral appliances were excluded. Poor quality cephalograms with artifacts that could interfere with the anatomical point identification were excluded as well. There was no restriction on patients’ gender, age, and ethnicity at the time that cephalometric X-rays were taken. A sample size calculation test was performed based on previous research. A minimum sample size of 79 patients was calculated as appropriate to detect a significant deviation in the intraclass correlation coefficient equal to or greater than 0.70 (moderate agreement and upwards) from 0.50 (poor agreement), with a power of 80%. A sample of 100 subjects was recruited and used in this project [23].

**Figure 1:**
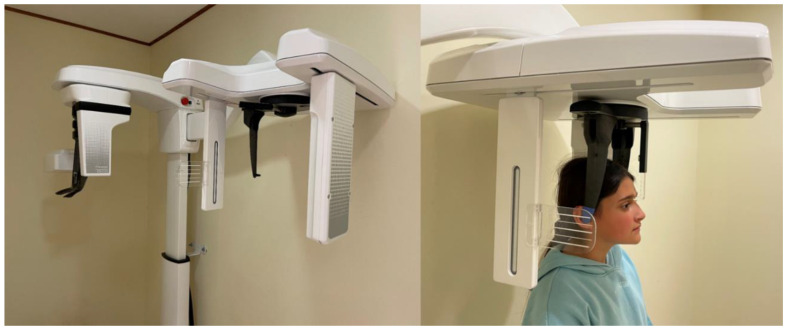
CS 8100 SC.

Pre-treatment lateral cephalometric radiographs of 100 patients (43 males, 57 females, mean age: 15.9 ± 4.8 years) were randomly selected. The cephalometric images were taken with the patient in the upright standing position with the Frankfort plane parallel to the floor, keeping the teeth in centric relation and the lips relaxed. All the lateral cephalometric radiographs were taken using the same lateral cephalometric machine (CS 8100 SC) by the same technician in the maximum image size (18 × 24 cm lateral image) (Figure 1).

**Figure 2:**
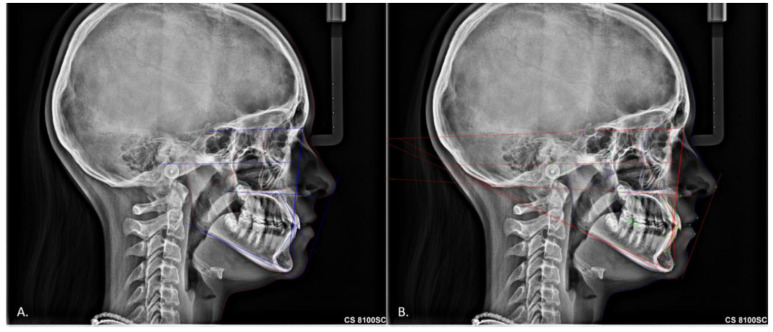
Cephalometric methods (**A**) Manual digital cephalometric tracing, (**B**) Automatic digital.

All 100 lateral cephalometric radiographs were imported first to Dolphin 3D Imaging program version 11.0 (Dolphin Imaging and Management Solutions, Chatsworth, CA, USA) for the digital manual cephalometric tracing, and afterward, the same lateral cephalometric radiographs were imported to CS imaging V8 (Carestream Dental LLC, Atlanta, GA, USA) for the digital automatic tracing (Figure 2). All manual tracings were made by a blinded experienced operator who is a diplomate of the American Boards of orthodontics (I.A.T.). The image’s name was randomly renamed, and the list was kept in an Excel spreadsheet. Cephalometric analysis was based on the American Board of Orthodontics analysis and the European Board of Orthodontics analysis. The ABO cephalometric measurements are SNA, SNB, ANB, SN-MP, FMA, U1-SN, U1-NA, L1-MP, and L1-NB. The EBO cephalometric measurements are SNA, SNPg, ANPg, SN/ANS-PNS, SN/Go-Gn, ANS-PNS/GoGn, U1/ANS-PNS, L1/GoGn, L1/APg, and U1-L1. This resulted in the identification of 16 cephalometric landmarks, which responded to 17 angular and 2 linear measurements.

### Statistical Analysis

All cephalometric measurement data were imported into an Excel spreadsheet (Microsoft, Redmond, WA, USA), and statistical analysis was performed using SPSS software (version 27; IBM, Armonk, NY, USA). Normal distribution of the data was tested using the Kolmogorov–Smirnov test. Descriptive statistics (mean, standard deviation, and minimum and maximum values) were calculated for every parameter measured by each method. Differences between methods were assessed using paired the *t*-test or Wilcoxon test, when appropriate. Furthermore, the Bonferroni method for multiple comparisons was applied for hypothesis testing of the equality of several parameters’ means between the automatic and manual methods. We applied the Bonferroni correction since we compared 18 parameters, so the level of statistical significance (a = 0.05) was divided by the number of parameters and was set to 0.0027 to avoid inflation of the type I error because of the multiple comparisons. The intra-method agreement was evaluated using the intraclass correlation coefficient (ICC) [26]. All comparisons were two-sided at a = 0.05 level of statistical significance.

## 3. Results

The sample included 100 subjects, 43 males and 57 females with a mean age of 15.9 ± 4.8 years. The operator’s reliability was calculated using intraclass correlation on 20 randomly selected subjects, whose data were re-measured 3 weeks apart. All measurements showed excellent intraclass correlation (Table 1).

### 3.1. American Board of Orthodontics Cephalometric Analysis

There was no significant difference between the two methods for the measurements of SNA, SNB, ANB, SN-MP, U1-SN, U1-NA, L1-MP, and L1-NB (*p* > 0.05) while there was a significant difference between the two methods for the measurements of FMA and L1-MP (*p* < 0.05). All measurements showed a high correlation between the two methods (ICC > 0.70) (Table 2 and Table 3).

### 3.2. European Board of Orthodontics Cephalometric Analysis

There was no significant difference between the two methods for the measurements of SNPg, ANPg, SN/ANS-PNS, SN/Go-Gn, U1/ANS-PNS, L1/GoGn, and L1/APg (*p* > 0.05) while there was a significant difference between the two methods for the measurements of ANS-PNS/GoGn and U1-L1 (*p* < 0.05). All measurements showed a high correlation between the two methods (ICC > 0.70) (Table 2 and Table 3).

## 4. Discussion

This study compared a digital automatic method and a digital manual method of the cephalometric analysis of the skull.

There have been studies that compared the accuracy of digital tracing to manually tracing X-rays on acetate paper at the very beginning of digital cephalometric analysis. Their findings revealed no statistically significant differences between the two strategies for identifying landmarks. There is a strong correlation between the reproducibility of landmarks within examiners when using manual and computerized procedures. However, whereas the measurement errors were generally equal, the inter-examiner repeatability of landmarks was unsatisfactory. Computerized measures offer a sizable time benefit over the manual approach. When the time benefits are considered, computer-assisted cephalometric studies can benefit physicians more because they do not result in an increase in intra- and inter-examiner error. The results of the digital cephalometric tracing between the various programs were identical [27,28,29].

Later, studies looked over the accuracy of automatic landmark identification for digital cephalometric analysis using different software. In 2020, Meric and Naoumova discovered that fully automated solutions can perform cephalometric analyses more quickly and accurately. According to the study’s findings, the manual correction of CephX landmarks produces results that are comparable to those of digital tracings made with CephNinja and Dolphin but take much less time to analyze. A year later, Bulatova et al. discovered that only the U apex, L apex, Basion, Orbitale, and Gonion landmarks identification from the automatic digital cephalometric approach revealed a statistically significant difference, but none of the other landmarks did [30,31].

Our study revealed significant differences in FMA, L1MP, ANS-PNS/GoGn, and U1-L1 landmarks while the rest showed no significant differences. These results showed an agreement with the research of Bulatova et al. It is important to mention that all measurements that resulted in a statistically significant difference between the two methods do not appear to have a clinically significant difference. In cephalometrics, for every measurement, there is a norm value with a standard deviation. The means and the standard deviation of the values between the manual and the automatic tracing that were statistically significant only differ in decimal points or by a couple of degrees. As a result, our final diagnosis will not be affected by these cephalometric measurements since those differences in the values are very small and will maintain a final diagnostic outcome in relation to the norms. Therefore, we can conclude that the automatic tracing method is reliable and accurate when used as a diagnostic method.

## 5. Conclusions

The automatic cephalometric tracing method using CS imaging V8 software is a reliable and accurate method for all cephalometric measurements. There was a high intraclass correlation coefficient between the two methods for all measurements. There were differences in FMA, L1-MP, ANS-PNS/GoGn, and U1-L1 measurements but they are not considered clinically significant.

## Figures and Tables

**Table 1:** Intraclass correlation coefficient (ICC) and 95% confidence interval (CI) for intra-method agreement.

Parameter	ICC (95% CI)	
	Automatic	Manual
ANB	1.00 (1.00, 1.00)	0.99 (0.97, 0.99)
ANPg	1.00 (1.00, 1.00)	0.99 (0.97, 0.99)
ANS-PNS/GoGn	1.00 (1.00, 1.00)	0.97 (0.93, 0.99)
FMA (MP-FH)	1.00 (1.00, 1.00)	1.00 (0.99, 1.00)
IMPA (L1-MP)	1.00 (1.00, 1.00)	0.98 (0.94, 0.99)
L1-NB	1.00 (1.00, 1.00)	0.97 (0.92, 0.99)
L1/APg	1.00 (1.00, 1.00)	1.00 (1.00, 1.00)
L1/GoGn	1.00 (1.00, 1.00)	0.98 (0.95, 0.99)
SN-GoGn	1.00 (1.00, 1.00)	0.98 (0.95, 0.99)
SN/ANS-PNS	1.00 (1.00, 1.00)	0.96 (0.89, 0.98)
SNA	1.00 (1.00, 1.00)	0.95 (0.88, 0.98)
SNB	1.00 (1.00, 1.00)	0.97 (0.92, 0.99)
SNMP	1.00 (1.00, 1.00)	0.98 (0.94, 0.99)
SNPg	1.00 (1.00, 1.00)	0.98 (0.96, 0.99)
U1-NA	1.00 (1.00, 1.00)	0.99 (0.99, 1.00)
U1-L1	1.00 (1.00, 1.00)	0.99 (0.99, 1.00)
U1-SN	1.00 (1.00, 1.00)	0.99 (0.99, 1.00)
U1/ANS-PNS	1.00 (1.00, 1.00)	1.00 (0.99, 1.00)

**Table 2:** Intraclass correlation coefficient (ICC) and 95% confidence interval (CI) for inter-method agreement (auto, manual).

Parameter	ICC (95% CI)
ANB	0.85 (0.70, 0.97)
ANPg	0.77 (0.61, 0.91)
ANS-PNS/GoGn	0.72 (0.45, 0.91)
FMA (MP-FH)	0.73 (0.47, 0.85)
IMPA (L1-MP)	0.70 (0.49, 0.87)
L1-NB	0.74 (0.76, 0.93)
L1/APg	0.75 (0.57, 0.89)
L1/GoGn	0.78 (0.60, 0.93)
SN-GoGn	0.89 (0.72, 0.92)
SN/ANS-PNS	0.77 (0.59, 0.93)
SNA	0.74 (0.55, 0.90)
SNB	0.78 (0.60, 0.94)
SNMP	0.89 (0.73, 0.93)
SNPg	0.92 (0.88, 0.94)
U1-NA	0.79 (0.70, 0.86)
U1-L1	0.70 (0.54, 0.81)
U1-SN	0.76 (0.61, 0.88)
U1/ANS-PNS	0.72 (0.53, 0.89)

**Table 3:** Descriptive statistics for each parameter depending on the method of measurement. Statistical significance set at *p* < 0.0027.

	Automatic			Manual			Analysis	
Parameter	Mean (SD)	min	max	Mean (SD)	min	max	Auto-Manual	*p*-Value *
ANB	3.8 (2.7)	0.0	14.0	3.6 (2.4)	0.1	9.5	0.2	0.517
ANPg	5.8 (5.0)	0.0	29.0	5.4 (3.8)	0.1	16.5	0.4	0.467
ANS-PNS/GoGn	27.1 (4.3)	17.7	45.0	24.1 (5.2)	9.4	33.8	3	<0.001 *
FMA (MP-FH)	30.2 (5.6)	15.9	49.0	28.1 (5.3)	14.9	39.4	2.1	<0.001 *
IMPA (L1-MP)	87.1 (7.6)	61.4	104.8	91.1 (7.8)	71.3	110.1	−4	<0.001 *
L1-NB	156.8 (16.1)	13.7	174.8	156.3 (6.6)	139.4	176.4	0.5	0.150
L1/APg	155.3 (27.0)	4.1	173.9	156.6 (15.4)	13.2	173.2	−1.3	0.945
L1/GoGn	90.6 (7.5)	64.1	107.0	93.2 (8.0)	74.2	112.1	−2.6	0.003
SN-GoGn	33.1 (6.6)	19.0	55.4	31.7 (5.4)	16.3	42.4	1.4	0.021
SN/ANS-PNS	7.0 (4.8)	0.0	18.8	7.7 (3.4)	1.1	17.0	−0.7	0.125
SNA	82.4 (6.5)	67.1	106.2	81.7 (3.4)	73.4	91.5	0.7	0.246
SNB	78.9 (6.5)	63.2	98.8	78.2 (3.8)	71.4	90.0	0.7	0.256
SNMP	35.6 (6.6)	21.7	58.5	34.4 (5.5)	19.5	45.4	1.2	0.061
SNPg	98.5 (7.8)	82.4	120.1	98.2 (7.9)	80.5	119.5	0.3	0.252
U1-NA	35.9 (5.5)	30.4	41.4	35.4 (3.4)	32	38.8	0.5	0.014
U1-L1	131.9 (11.1)	107.0	167.6	128.6 (10.5)	101.2	157.0	3.3	<0.001 *
U1-SN	105.6 (7.7)	88.2	122.6	105.9 (8.0)	90.2	124.6	−0.3	0.629
U1/ANS-PNS	111.6 (5.5)	96.4	125.8	112.8 (9.2)	54.2	130.9	−1.2	0.195

* it is used broadly for statistical significance results.

## Data Availability

Not applicable.
